# Storage Temperature or Thermal Treatments During Long Egg Storage Duration Influences Hatching Performance and Chick Quality

**DOI:** 10.3389/fphys.2022.852733

**Published:** 2022-03-01

**Authors:** Maryse Guinebretière, Julie Puterflam, Alassane Keïta, Sophie Réhault-Godbert, Rodolphe Thomas, Pascal Chartrin, Estelle Cailleau-Audouin, Edouard Coudert, Anne Collin

**Affiliations:** ^1^Epidemiology, Health and Welfare Unit, Ploufragan-Plouzané-Niort Laboratory, French Agency for Food, Environmental and Occupational Health and Safety (ANSES), Ploufragan, France; ^2^Technical Institute for Poultry (ITAVI), Ploufragan, France; ^3^INRAE, Université de Tours, Nouzilly, France

**Keywords:** breeder flock age, storage conditions, embryonic development, embryo mortality, hatchability, chick quality, broiler performance, antioxidant status

## Abstract

This study was designed to improve the hatching performance, chick robustness and poultry health in the event of long-term egg storage and suboptimal age of the reproductive flock. A total of 9,600 eggs from one young breeder flock (28 weeks of age, batch B) and 9,600 eggs from an older breeder flock (59 weeks of age, batch E) were used (ROSS 308). Each batch was separated into three sub-groups and stored for 14 days. The first sub-group of eggs (Cool, group C) was stored at 11.6°C. The second sub-group of eggs (Warm, group W) was stored at 18.3°C with two pre-incubation on days 6 and 10 of the storage period. The final sub-group of eggs (Control, group Ct) was stored at 18.3°C throughout the storage period. Eggs were similarly incubated and hatched birds were raised on the same experimental farm. In both batches, embryonic development was significantly more advanced in W eggs than in C and Ct eggs ( *p* < 0.01). In both batches, C and W treatments decreased early embryonic mortality by more than 10% compared with Ct, decreased the proportion of late-hatched chicks and improved the percentage of first grade chicks: in batch E, 42% of Ct eggs were first grade chicks vs. 57% in group W and 59% in group C. Benefits were even higher in batch B, where only 60% of Ct eggs gave first grade chicks vs. 83% in others groups. The hatching rate was thus higher in groups C and W regardless of flock age: for batch B eggs, 85% hatched in W and 84% in C vs. 62% in Ct, while for batch E eggs, 59% hatched in W and 61% in C vs. 45% in Ct. Day-old Ct chicks from batch E were heavier than W and C ones, and heavier than W chicks from batch B ( *p* < 0.05). Long-term parameters on farm were not significantly different between groups. Thermal treatments during the storage of eggs from both young and old breeder flocks counterbalance the negative effects of prolonged egg storage on hatching rate, without altering chicken performance during rearing.

## Introduction

In order to coordinate hatchery activities and improve their flexibility to meet market fluctuations and demand, the duration of egg storage may vary in the broiler breeder industry. Hatcheries may have to extend this storage period up to 15 days to allow the incubation of thousands of eggs, and accordingly to obtain chickens of the same age in sufficient quantities. Hatcheries are also dependent on the availability of breeder flocks to meet demands for day-old chicks, with either young flocks (before 35 weeks of age) or older ones (over 55 weeks of age). However, neither the egg storage period nor the breeder flocks’ age are always optimised in commercial situations, with potentially detrimental effects on egg quality, incubation efficiency, the quality of chicks and post-hatching performance.

Prolonged egg storage (7 days or more) has negative consequences on embryonic survival, the hatching rates of fertile eggs and chick quality (Lapão et al., 1999; Tona et al., 2003a,b; Fasenko, 2007; Hamidu et al., 2010). It can also have a negative effect on the long-term performance of chickens (body and carcass weight; Alsobayel and Al-Miman, 2010). Moreover, hatchability increases when breeder flocks are aged less than 40 weeks old (Abiola et al., 2008) and then decreases, in part due to increased total embryo mortality (Almeida et al., 2008) and lower fertility (Guise et al., 1998). Breeder flock age also affects egg weight (Tona et al., 2001) and therefore influences chick weight (Tanure et al., 2009) at hatching: the eggs are generally heavier if they come from an older flock than from younger flocks. The quality of chicks from a young flock appears to be better (Tona et al., 2004) and their mortality rate is lower (Peebles et al., 1999).

The duration of egg storage and the age of the breeder flock often interact (Nasri et al., 2020). The age of the flock is likely to play a major role in the embryo’s ability to withstand prolonged storage periods; eggs from young breeder flocks can be stored longer without deterioration than eggs from older flocks (Reijrink et al., 2009).

Storage conditions can have a dramatic impact on hatchability (reviewed in Bergoug et al., 2013a). In commercial hatcheries, when eggs are stored for up to 1 week, they are generally kept at a constant temperature of 18°C–20°C and a relative humidity (RH) of 70–75% (Christensen et al., 2002). To help reduce the negative effects of prolonged storage, temperature and RH are empirically adjusted.

One solution is to decrease the storage temperature below physiological zero (Edwards, 1902 or embryonic diapause Fasenko, 2007), i.e. 20–21°C. Generally, the temperature in the storage room is decreased to 15–16°C as recommended by several authors (Christensen et al., 2002; Elibol et al., 2002). Under the influence of hypothermia, embryogenesis pauses, without any embryonic development: some cellular metabolic processes continue, but gross morphological (shape and structural) changes are stopped. Lowering temperature even more could also be beneficial. For instance, Pokhrel et al. (2018) compared egg storage for 7–28 days at either 18°C or 12°C with respect to hatchability and chick quality, and suggested lowering the storage temperature to 12°C for extended storage (beyond 7 days). Ruiz and Lunam (2002) also obtained better results for hatching rate and chick weight at hatching using eggs stored for 9–11 days at 10°C than at 16.5°C. However, according to Reijrink et al. (2008), this solution might not be enough for longer egg storage periods, since there is still an increase in the number of necrotic cellular indicators contributing to the early mortality of embryos. To date, very few publications about the impact of low temperature (under 12°C) on hatchability and chick quality are available.

Another solution is the pre-incubation of eggs, i.e. raising the temperature to 37°C for six continuous hours or several times during storage. Several authors have investigated this solution with respect to hatchability and chick quality (reviewed in Reijrink et al., 2008). The objective of this strategy is to synchronise the embryos to attain a more advanced stage of development that is less sensitive to prolonged storage (Pokhrel et al., 2017). The embryo germ cells could thus be in a better condition and the viscosity of the subgerminal liquid be maintained. However, the results obtained appear variable: hatching rate increased and embryonic mortality decreased when eggs were warmed for 6 h at 37°C (Silva et al., 2008), whereas another study (Fasenko et al., 2003) found adverse effects on the hatching rate by using this technique a few days after oviposition. Dymond et al. (2013) found better hatchability, lower embryo mortality and higher body weight over the 4 weeks post-hatching after several periods of pre-incubation of eggs every 4–5 days of storage, whereas Silva et al. (2008) advise against exposing eggs from old breeding flocks to a higher temperature (36°C) for extended periods of storage.

Pre-incubation appeared to be particularly important for embryos from young hens (Reijrink et al., 2009) but was not clearly demonstrated by Damaziak et al. (2018), who tested two pre-incubation periods at 5 and 10 days over 12 days of egg storage. It seems that the beneficial effect of pre-incubation depends on the interaction between several factors, such as the developmental stage of the embryo at oviposition, the length of storage, the length of the pre-incubation period and the number of times when it is applied (Reijrink et al., 2008). Finally, the relationship with the breeder flock’s age is not fully clear as related studies are sometimes contradictory.

It has been well established that hatchability declines with storage duration and the breeder flock’s age. However, the medium- and long-term consequences of cool or warm treatments prior to egg incubation have not been fully explored, especially those on animal health during rearing. The present research was designed to explore how to compensate for the negative impacts of long storage and the suboptimal age of breeder flocks in order to increase the hatching performance without deteriorating chick robustness, and improving poultry health throughout the rearing phase. The goal of this study was thus to measure the short-, medium- and long-term effects of different temperature conditions during storage when eggs, collected from young or old breeder flocks, are stored for 14 days. In one case, eggs were kept at low temperatures (11.6°C). In another, eggs were kept at 18.3°C with two successive periods of short pre-incubation thermal exposures. Egg quality (pH, weight loss, embryo mortality, embryo staging and hatchability), chick quality (proportion of first grade, vitellus and body weight) and broilers’ health and performance up to slaughter (mortality, body weight, breast yield and oxidative balance in the blood) were compared with eggs stored under conventional conditions (18.3°C, RH 69%).

## Materials and Methods

### Animals and General Husbandry

#### Egg Origin

Two batches of 9,600 eggs from two separate ROSS 308 breeding flocks, one at the beginning and the other at the end of their laying period (B: 28 weeks of age, E: 59 weeks of age), were used (September 2018). All the eggs were purchased from the Perrot hatchery (Pommerit-Jaudy, France). They were then considered separately and designated as batches B and E.

#### Egg Storage

**Figure 1:**
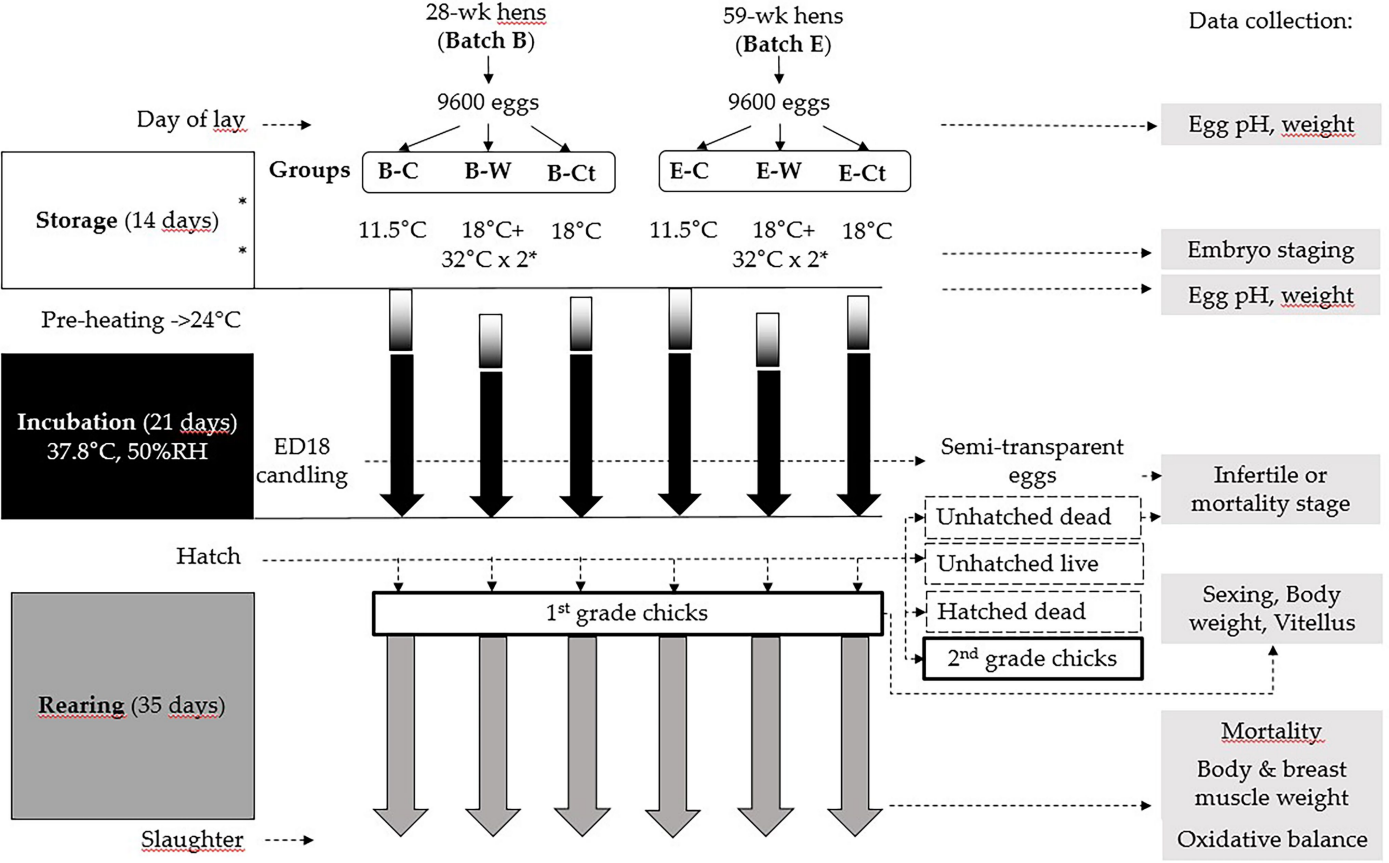
Experiment workflow (B: eggs from breeder flock at the beginning of the laying period and E: end of the laying period). Ct: control group; C: cool storage; and W: warm storage. ^*^pre-incubations (for groups W), dotted box: unhatched products, bold box: hatched chicks.

All the eggs from both batches were stored for 14 days at the ANSES Ploufragan experimental facilities (France) according to three modes, forming three groups of 3200 eggs: control group (Ct), cool group (C) and warm group (W). The average temperature of the Ct group was 18.3°C (18.3–18.4), with RH 69.4% (69.3–69.5) for the entire storage period. C eggs were stored in a cool room, the target being 11°C for the entire storage period. Actually, the average T° was 11.6°C (11.6–11.7) and RH 70.1% (69.6–70.5). Finally, W eggs were stored in the same room as Ct eggs, the difference being that they were warmed twice in incubators, on day 6 and day 10 after oviposition. During these two pre-incubations, the temperature reached a plateau of 34°C in 3.5 h, then remained at 34°C for 2 h before going back down under 26°C in 2 h. The total duration above 32°C was 4 h. The experiment workflow is summarised in Figure 1.

#### Incubation Period

After the 14 days of storage, all the eggs were incubated in the experimental hatchery using two incubators. One incubator was used for B eggs, another one for E eggs (Incubators/Hatchers, 9,600 egg capacity, Petersime^®^, Zulte, Belgium). The three groups were homogeneously distributed in each incubator. The incubation parameters (temperature, CO_2_ and hygrometry) were in accordance with the manufacturer’s recommendations.

Before incubation started, the eggs were pre-heated for different lengths of time depending on the group, slowly reaching 24°C to limit condensation on the eggshells, especially for C eggs. Thus, C eggs were pre-heated longer (12 h) than the other eggs (8 h). In addition, hatching time was supposed to be shorter for W eggs (Dymond et al., 2013). In order to have all the eggs hatching at the same time, the incubation of W eggs was therefore delayed by 8 h compared with Ct and C eggs.

In each group, eggs were candled after 18 days of incubation. Semi-transparent eggs were discarded. The others were put back into the incubators until hatching.

#### Hatching

At 21 days of incubation (523 h for Ct and C eggs; 515 h for W eggs), the results of incubation were divided into either unhatched products or hatched chicks.

Unhatched products were further divided into:

- Unhatched dead in shell embryos,
- Unhatched live in shell chicks at internal/external pipping, and
- Hatched dead chicks outside the shell.

Based on physical parameters, hatched chicks were blindly classified by trained staff as:

- First grade chicks: free of any visible abnormality, dry and clean, active and bright eyes (Tona et al., 2004).
- Second grade chicks: either inactive or with an abnormality or defect in the navel and legs/feet. They included in this category wet chicks, considered to be late-hatched as: these chicks were alive and outside of the eggshell, but although fully hatched their fuzz was still wet.

In each group, the first grade chicks were sexed (vent sexing).

#### Rearing Period

First grade chicks were placed directly next to the experimental farm’s hatchery. One room housed B chicks, and another, E chicks. In both rooms, one side housed males and the other side housed females, which were separated by a central corridor. In each batch × sex block, the three groups were homogeneously distributed in each side, in pens with a density of 19 chicks/m^2^. All the animals were reared under the same conditions: the rearing parameters (feeding, lighting programme, temperature, ventilation and vaccination) followed the classic standard commercial specifications (AVIAGEN, 2014). All animals had *ad libitum* access to water and feed. Animals were slaughtered in a commercial slaughterhouse at 35 days of age. Depending on the numbers of hatched chicks, 1,274–2,381 chickens were reared in each group. There were 3–6 pens of 170 animals per group × sex block.

### Data Collection

Unless specified, all the following measurements were taken for each batch.

#### Egg Parameters During Storage

Before and after the storage period, 50 randomly selected eggs per group were weighed (0.1 g precision) to estimate weight loss, and 20 eggs were broken to measure egg white pH (HI 9321 microprocessor pH meter, HANNA Instruments, Lingo, France).

**Figure 2:**
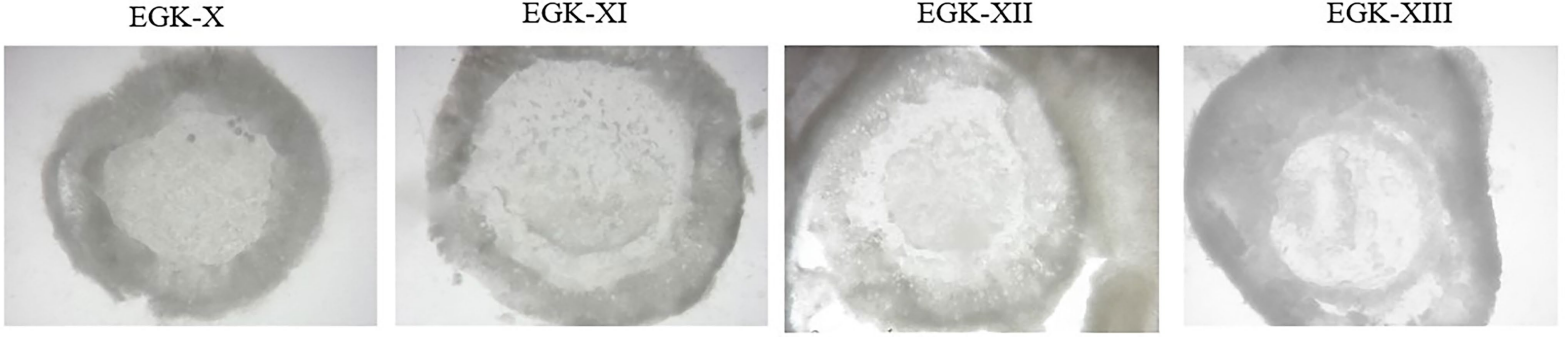
Ventral view of the blastoderm from stages EGX to EGXIII—^©^ANSES.

After 12 days of storage, 15 eggs per group were opened to determine the embryo staging according to Eyal-Giladi and Kochav classification (Eyal-Giladi and Kochav, 1976). Specific methods for embryo isolation and staging were described by Pokhrel et al. (2017) and Wade et al. (2014). Briefly, forceps were used to open the blunt end of the egg, exposing the underlying air space. Albumen was removed from the yolk, and the embryo was removed from the egg by placing a filter paper disc containing a 1-cm hole in the centre, over the embryo. Dissection scissors were used to cut around the disc through the perivitelline layer. The disc containing the germinal disc embryo was removed and cleaned in PBS (pH 7.4) to remove any adherent yolk. The embryos were placed in Petri dishes and observed using a dissection microscope (Nikon SMZ1500, Melville, NY). Stage EGX was defined by completion of area pellucida formation (Figure 2). At this stage, polyingressing cells and cell clusters were visible on the ventral surface of the area pellucida. Notably, at stage EGX, no hypoblast cells in the posterior part of the embryo, adjacent to Koller’s sickle, had formed. In the following stages, EGXI-XIII, the hypoblast formed in a posterior to anterior direction; at stage EGXI, the hypoblast covered one-third of the area pellucida; at stage EGXII, it covered two-thirds of the area pellucida; and by stage EGXIII, hypoblast formation was complete.

#### Hatching Parameters

The number of semi-transparent eggs defined at 18 days of incubation was counted in each group and was related to the initial number of eggs in the incubator (Ni). The total number of unhatched eggs at 21 days of incubation was counted in each group and was related to Ni or to the number of eggs kept in the incubator after the candling process (Ns). Semi-transparent eggs (at 18 days of incubation) and unhatched eggs (at 21 days of incubation) were cracked open immediately after having been sorted and were visually analysed to determine infertility or embryo mortality staging:

- Stage 0: no visible embryo. The egg was considered as infertile or fertile but in which the embryo died before 4 days of incubation.
- Stage 1: embryo died before 4 days of incubation: the germinal disc was visible.
- Stage 2: embryo died between 4 and 6 days of incubation: presence of a blood network.
- Stage 3: embryo died between 7 and 17 days of incubation: presence of black eyes.
- Stage 4: embryo died after 18 days of incubation: growth of embryo nearly complete and yolk sac drawn into body cavity.

In each group, the number of eggs in each stage was related to Ni.

The number of dead chicks and the number of unhatched chicks were counted for each group and related to Ni or to Ns.

The number of first grade chicks and second grade chicks were counted for each group and related to Ni or to the total number of hatched chicks (Nh). This was also considered as the hatching rate. The number of wet chicks was counted for each group and related to Ni or Nh. The total number of wet chicks plus unhatched chicks was counted for each group and related to Ni or to Ns.

#### Measurements on First Grade Chicks

The sex ratio of first grade chicks was determined in each group (number of females related to the number of first grade chicks). In each group, 100 chicks (50 males and 50 females) were weighed and 40 chicks (20 male and 20 females) were euthanised and their vitellus weighed, then related to the chick’s total body weight.

#### Measurements on Broilers During Rearing

##### Mortality During the Rearing Period

During the rearing period, mortalities were recorded daily. For ethical reasons, birds that had severe health problems (i.e. fracture, blindness, severe lameness and malformation) were culled and recorded as dead. The cumulated mortality rates on days 5, 11, 14 and 35 were calculated as the cumulated number of dead animals to the initial number of animals on the farm.

##### Body Weight During the Rearing Period

In each group, 200 animals (100 males and 100 females) were weighed at 4, 11, 21, 28 and 32 days of age. Animals were randomly selected in each group. The individuals weighed in each group were thus potentially different or identical between each weighing session.

##### Breast Muscle Weight

In each group, 16 randomly selected broilers (eight males and eight females) were euthanised at 31 days (for E) or 32 days (for B). Their right muscle breast (pectoralis major) was weighed. The weight was multiplied by two and related to the bird’s total body weight.

##### Oxidative Balance in the Blood of E Animals

Thermal treatments during egg storage could have an incidence on the antioxidative properties of chicks. These changes in oxidative balance may in turn have an impact on poultry health and even resistance to disease or other stresses. No research has been carried out in this area until now. An exploratory study was therefore performed to investigate the oxidative balance using several potential markers of oxidative stress. Markers of oxidation and antioxidant mechanisms were investigated in poultry from batch E at 7 and 31 days old. No poultry from batch B was included in this exploratory study. E birds were chosen based on the hypothesis that they would be more impacted by a long egg storage period.

At 7 and 31 days old, blood samples were taken from 16 broilers per E group (eight males and eight females). Blood was sampled from the occipital sinus. Whole blood was centrifuged at 3,000 *g* for 10 min, and plasma samples were stored at −80°C for further analysis of oxidative stress and antioxidant mechanisms. The thiobarbituric acid reactive substance (TBARS) index, commonly used to assess the susceptibility of tissues to peroxidation (Lin et al., 2004; Alnahhas et al., 2015), was measured in plasma samples according to Lynch and Frei (1993). The antioxidant status was assessed in plasma by measuring total antioxidant status (TAS; Pertusa et al., 2017) by using the Randox TAS colorimetric assay according to the supplier’s recommendations and using an ARENA 20XT (Thermo Scientific) device. Plasma concentrations of glucose, uric acid (a marker of protein oxidation for energy purposes, but also a potent antioxidant) and triglycerides were measured using miniaturised commercial kits (BioMérieux, Craponne, France) and a clinical chemistry analyser (ARENA, Thermo Fisher Scientific, Courtaboeuf, France).

### Statistical Methods

The statistical analysis was conducted using R software (R Core Team, 2020). The B and E batches are considered as two distinct batches, so they were analysed separately and they were not compared between themselves. Indeed, although conditions of storage were perfectly similar between the two batches, eggs came from different farms, different breeders flocks, which can strongly influence egg quality, egg fertility and thus hatching rate, and chick quality. Moreover, incubation and rearing were done in separate incubators and rearing rooms.

After checking the normality of the data by a Shapiro–Wilk test and the equality of variances by a Bartlett test, egg weight loss, pH and embryo staging after the 14-day storage period were examined by an analysis of variance, taking into account the group as explanatory variables, followed by a Student–Newman–Keuls test as *post-hoc* analysis. Chick weight, vitellus weight, body weight during rearing and breast muscle weight were all examined using an analysis of variance taking into account the group and sex as explanatory variables and their interactions, followed by a Student–Newman–Keuls test.

The results of hatching parameters, embryo mortality stages in relation to Ni, Ns or Nh, sex ratio relative to first grade chicks and mortality rates on days 5, 11, 14 and 35 were analysed using Chi-square tests.

The results for oxidative balance in the blood were examined by an analysis of variance taking into account the age of the animals, the group and the sex as explanatory variables and their interactions, followed by a Student–Newman–Keuls test as *post-hoc* analysis.

For all analyses, differences were considered significant when *p* < 0.05.

### Ethics Approval

All research was reviewed and approved by the Comité National de Réflexion Ethique sur l’Expérimentation Animale (National committee for ethics in animal testing) No. 016 at the French Ministry for Education, Higher Education and Research before data collection (number APAFIS#16128–2,018,071,314,183,633 v1). All efforts were made to minimise the number of animals used and their suffering.

## Results

### Eggs

The results of egg weight loss, albumen pH and embryo staging after a 14-day storage period are shown in Table 1.

**Table 1:** Mean (SD) of weight loss (% of initial weight), albumen pH and embryo staging (EG&K classification) after 14 days of storage.

	**B batch**				
	**C**	**W**	**Ct**	**P Group**	
Weight loss	1.0 (0.3)^b^	1.9 (0.5)^a^	1.7 (0.3)^a^	<0.001	
Albumen pH	8.7 (0.03)^c^	8.9 (0.04)^a^	8.8 (0.04)^b^	<0.001	
Embryo staging	10.0 (0.00)^b^	12.4 (1.04)^a^	10.1 (0.67)^b^	<0.001	
	**E batch**				
	**C**	**W**	**Ct**	**P Group**
Weight loss	1.1 (0.5)^b^	1.8 (0.4)^a^	1.7 (0.3)^a^	<0.001
Albumen pH	8.8 (0.04)^c^	9.0 (0.04)^a^	8.9 (0.05)^b^	<0.001
Embryo staging	10.4 (0.70)^b^	12.6 (1.09)^a^	10.2 (0.63)^b^	<0.001

Ct, control group; C, cool storage; and W, warm storage. B: eggs from breeder flocks at the beginning of the laying period; E: end of the laying period. n per group = 50 eggs (weight loss), 20 eggs (pH) and 15 eggs (embryo staging). p indicates the statistical difference between groups within batches. a, b and c: values not associated with common letters within batches are statistically different (*p* < 0.05).

Before storage, pH values were 8.5 and 8.4 in B and E eggs, respectively. After 14 days of storage, in both B and E batches, the pH was higher for W eggs than for Ct eggs and was lower for C eggs than for Ct eggs.

Before storage, embryo staging was at 10.2 and 10.7 in B and E eggs, respectively. After 14 days of storage, in both batches B and E, the developmental stage of the embryo was higher in W than in Ct eggs, while Ct and C embryo stages were not significantly different.

### Hatching Results

Table 2 summarises the hatching results per group in batches B and E, and the results of the statistical comparison of proportions relative to Ni.

**Table 2:** Number of incubated eggs (Ni), semi-transparent eggs eliminated at ED18, eggs kept in the incubator after the candling process (Ns), unhatched products—detailed as unhatched dead in shell embryos, hatched dead chicks and unhatched live in shell chicks; number of hatched chicks (Nh)—detailed as first and second grade chicks including wet chicks.

		**B batch**				**E batch**			
		**C**	**W**	**Ct**	***p***	**C**	**W**	**Ct**	***p***
Incubated eggs (Ni)		3200	3200	3200	–	3200	3200	3200	–
Semi-transparent eggs		297^b^	285^b^	788^a^	<0.001	1024^b^	1036^b^	1384^a^	<0.001
Viable eggs (Ns)		2903^b^	2915^b^	2412^a^	<0.001	2176^b^	2164^b^	1816^a^	<0.001
Unhatched products	Unhatched dead	104^b^	74^c^	175^a^	<0.001	158^b^	173^b^	229^a^	<0.001
	Hatched dead	36	41	44	0.663	38	52	37	0.186
	Unhatched live	74^b^	67^b^	196^a^	<0.001	39^b^	41^b^	96^a^	<0.001
Hatchlings (Nh)		2689^b^	2733^b^	1997^a^	<0.001	1941^b^	1898^b^	1454^a^	<0.001
1st grade chicks	1st grade chicks	2649^b^	2666^b^	1919^a^	<0.001	1889^b^	1830^b^	1358^a^	<0.001
	*males*	*1350*	*1320*	*1086*	–	*929*	*932*	*677*	–
	*females*	*1299*	*1346*	*833*	–	*960*	*898*	*681*	–
2nd grade chicks	2nd grade chicks	40^b^	67^a^	78^a^	0.002	52^b^	68^b^	96^a^	<0.001
	*wet chicks*	15^b^	21^b^	60^a^	<0.001	21^b^	18^b^	36^a^	0.024

Ct, control group; C, cool storage; and W, warm storage. B: eggs from breeder flocks at the beginning of the laying period; E: end of the laying period. p indicates the statistical difference between groups within batches, comparing numbers relative to Ni. a, b: values not associated with common letters within batches are statistically different (*p* < 0.05).

For both B and E batches, the number of semi-transparent eggs (at ED18) relative to Ni was higher in Ct groups than in either C or W groups. They represented 25% of Ni eggs in the B-Ct group, whereas they represented less than 10% in the B-C and B-W groups. Semi-transparent eggs represented 43% of Ni eggs in the E-Ct group, whereas they were less than 33% in both E-C and E-W groups.

Similarly, for both B and E batches, there were more unhatched dead in shell (at E21) relative to Ni (or to Ns) in Ct groups than in C or W groups. Unhatched dead in shell represented 7.1% of Ni eggs in the E-Ct group, whereas they represented less than 5.5% in both E-C and E-W groups. In the B-W group, the number of unhatched dead in shell was even lower than in the C group: unhatched dead in shell represented 2.3% of Ni eggs in the B-W group, whereas it was 3.2% in group B-C and 5.5% in B-Ct.

The number of hatched dead chicks outside the shell was less than 1.6% of Ni in all groups, regardless of the batch (B or E) or the group (C, W or Ct).

**Figure 3:**
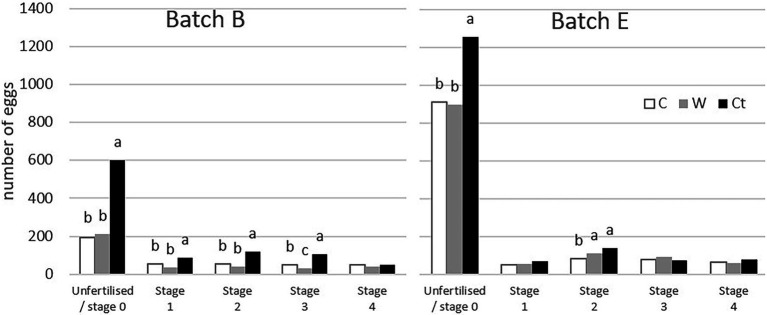
Mortality stage of embryos from unhatched eggs (at ED21) and semi-transparent eggs (at ED18; B: eggs from breeder flock at the beginning of the laying period and E: end of the laying period). Ct: control group; C: cool storage; and W: warm storage. a, b and c: values not associated with common letters within stages are statistically different at threshold *p* < 0.05 [comparing numbers relative to the number of incubated eggs (Ni)].

The details of mortality stages in semi-transparent eggs and unhatched eggs are shown in Figure 3. Ct groups contained more stage 0 than other groups, in both B and E batches. They represented 18.8% of Ni eggs in the B-Ct group but less than 7% in the B-C and B-W groups. They represented 39.3% of Ni eggs in the E-Ct group but less than 29% in both the E-C and E-W groups.

Mortality stages 1, 2 and 3 decreased in the B-C and B-W groups in comparison with the B-Ct group. This was only the case for mortality stage 2 in batch E for the C group. The higher number of stage 0 in the Ct groups strongly affected the Ns that had lower values in the Ct group than in either the W or C groups in both B and E batches (Table 2).

For both B and E batches, Nh (or hatching rate) was lower in the Ct groups than in either C or W relative to Ni or Ns. The hatching rate increased from 62.4% of Ni in B-Ct to 85.4 and 84.0% in the B-W and B-C groups, respectively, and from 45.4% in E-Ct to 59.3 and 60.7% in the E-W and E-C groups, respectively.

For both B and E batches, the relative number of first grade chicks was lower in the Ct groups than in either C or W. In addition, the proportion of first grade chicks relative to Nh was even higher in the B-C group than in the B-W group: 96.1% of Nh in B-Ct were first grade, 97.5% in B-W and 98.5% in B-C, *p* < 0.001 for each pairwise comparison. Conversely, the proportion of second grade chicks relative to Nh was higher in B-Ct groups than in B-W groups, the latter being higher than in B-C groups (3.9, 2.5 and 1.5% in B-Ct, B-W and B-C groups, respectively, *p* < 0.001 for each pairwise comparison).

In batch E, 93.4% of Nh in E-Ct were first grade, 96.4% in E-W and 97.3% in E-C groups (there was no significant difference between the E-W and E-C groups; E-Ct was lower than other groups, p < 0.001). Conversely, the proportion of second grade chicks relative to Nh was higher in the E-Ct groups than in E-C and E-W, without any significant difference between these two groups.

To sum up, 42.4% of group E-Ct eggs became first grade chicks vs. 57.2% in E-W and 59.0% in E-C. The benefits of cool or warm storage conditions were even higher in batch B where only 60.0% of eggs in group B-Ct were first grade chicks vs. 83.3% in B-W and 82.8% in B-C, respectively.

For both B and E batches, the relative number of wet chicks was higher in the Ct groups than the C or W groups. This was also the case for the number of unhatched chicks (higher in the Ct groups than in the C or W groups), which can also be considered as late-hatched chicks (live chicks but not fully hatched). These two categories taken together represented, respectively, 3.1, 3.0 and 10.6% of B-C, B-W and B-Ct eggs relative to Ns, while they represented 2.8, 2.7 and 7.3% of E-C, E-W and E-Ct Ns eggs, respectively.

### First Grade Chicks

#### Sex Ratio

Females were fewer in the E-Ct group (43.4%, Table 2). This sex ratio was significantly different from that of groups E-W (50.5%, *p* < 0.001) and E-C (49.0%, *p* < 0.001). For batch E, there was no difference in the sex ratios of first grade chicks between groups (females represented 50.8, 49.1 and 50.1% of E-C, E-W and E-Ct chicks, respectively, *p* = 0.562).

#### Chick Weight and Vitellus Weight

Body weight and vitellus weight are shown in Table 3.

**Table 3:** Mean (SD) of first grade chick weight (g; *n* = 50 chicks, by sex per group) and percentage of vitellus weight related to body weight (*n* = 20 chicks, by sex per group).

	**B batch**					
	**C**	**W**	**Ct**	**P Group**	**P Sex**	**P Group × Sex**
Chick weight	36.5 (2.9)^a^	35.6 (2.2)^b^	36.7 (2.5)^a^	0.0095	0.004	0.542
Vitellus %	8.0 (2.0)	7.5 (1.8)	8.3 (2.0)	0.169	0.847	0.626
	**E batch**					
	**C**	**W**	**Ct**	**P Group**	**P Sex**	**P Group × Sex**
Chick weight	45.0 (3.6)^b^	45.0 (3.6)^b^	47.0 (3.3)^a^	<0.001	0.037	0.096
Vitellus %	8.3 (3.4)^b^	8.5 (2.5)^b^	10.4 (4.0)^a^	0.011	0.019	0.656

Ct, control group; C, cool storage; and W, warm storage. B: chicks from breeder flocks at the beginning of the laying period; E: end of the laying period. p indicates the statistical difference between groups within batches; a, b and c: values not associated with common letters within batches are statistically different (*p* < 0.05).

B-W chicks were lighter than B-Ct and B-C groups, with no difference between B-C and B-Ct chick weights. There was no difference in the vitellus/weight ratio between groups. E-W and E-C chicks were lighter than those from the E-Ct group, and both males and females had a relatively lighter vitellus.

In both B and E batches, females were heavier than males (36.7 vs. 35.8 g in batch B female and male chicks, respectively, and 46.2 vs. 45.2 g in batch E female and male chicks, respectively). In batch E chicks, females had a higher vitellus/weight ratio than males (9.8% vs. 8.4%).

### Broilers During Rearing

#### Mortality During the Rearing Period

Mortality during rearing did not exceed 3% in any of the E batch groups. In batch B, mortality was less than 3% on the fifth day of age across all groups and increased to 4.2% by the end of the rearing period. No significant difference in mortality rates was observed among groups for both B and E broilers, or between males and females at any age.

#### Body Weight During the Rearing Period and Breast Muscle Weight

Table 4 shows the mean body weights during rearing and the relative breast muscle weight at the end of the rearing period for each group.

**Table 4:** Mean (SD) of body weight (BW, g) at 4, 11, 21, 28 and 32 days of age and relative breast muscle weight (pectoralis major PM, % in relation to body weight) at 31 (E) and 32 (B) days of age.

	**B batch**							
	**C**	**W**	**Ct**	**Male**	**Female**	**P Group**	**P Sex**	**P Group × Sex**
**BW 4**	72 (7)^b^	73 (6)^b^	74 (7)^a^	73 (6)	73 (7)	0.005	0.675	0.161
**BW 11**	281 (23)	281 (26)	280 (24)	285 (26)	276 (22)	0.801	<0.001	0.300
**BW 21**	884 (77)	891 (74)	897 (72)	929 (68)	852 (60)	0.251	<0.001	0.835
**BW 28**	1494 (161)	1513 (147)	1519 (159)	1618 (118)	1396 (95)	0.272	<0.001	0.358
**BW 32**	1925 (239)	1918 (183)	1923 (220)	2078 (153)	1765 (129)	0.940	<0.001	0.254
**PM**	18.4 (1.3)	18.6 (1.1)	18.0 (1.2)	18.4 (1.2)	18.4 (1.2)	0.287	0.946	0.636
	**E batch**							
	**C**	**W**	**Ct**	**Male**	**Female**	**P Group**	**P Sex**	**P Group × Sex**
**BW 4**	89 (7)	89 (7)	90 (8)	89 (7)	89 (7)	0.311	0.152	0.563
**BW 11**	315 (24)	316 (24)	315 (23)	322 (24)	310 (22)	0.983	<0.001	0.583
**BW 21**	962 (85)	956 (90)	965 (80)	1,022 (73)	915 (62)	0.244	<0.001	0.251
**BW 28**	1632 (182)	1610 (166)	1591 (166)	1759 (139)	1507 (106)	0.130	<0.001	0.244
**BW 32**	2037 (235)	1994 (247)	1987 (233)	2,198 (196)	1856 (140)	0.749	<0.001	0.951
**PM**	18.0 (1.2)	17.6 (1.2)	17.9 (1.1)	17.6 (1.4)	18.0 (1.0)	0.628	0.263	0.788

Ct: control group; C: cool storage; and W: warm storage. B: chickens from breeder flocks at the beginning of the laying period; E: end of the laying period. n, by sex per group = 100 animals (body weight), eight animals (PM). *p* indicates the statistical difference between groups within batches; a and b: values not associated with common letters within batches are statistically different (*p* < 0.05).

From 11 days of age onwards, males were heavier than females in all groups. No difference in body weight was observed among the groups, at any age except 4 days of age, when B-Ct chicks were heavier than both B-C and B-W chicks.

No difference in relative breast muscle weight was observed between groups (*p* = 0.628) and sexes (*p* = 0.263).

#### Markers of Oxidation and Antioxidant Mechanisms in Blood

##### Oxidative Stress and Antioxidant Markers in Plasma

**Figure 4:**
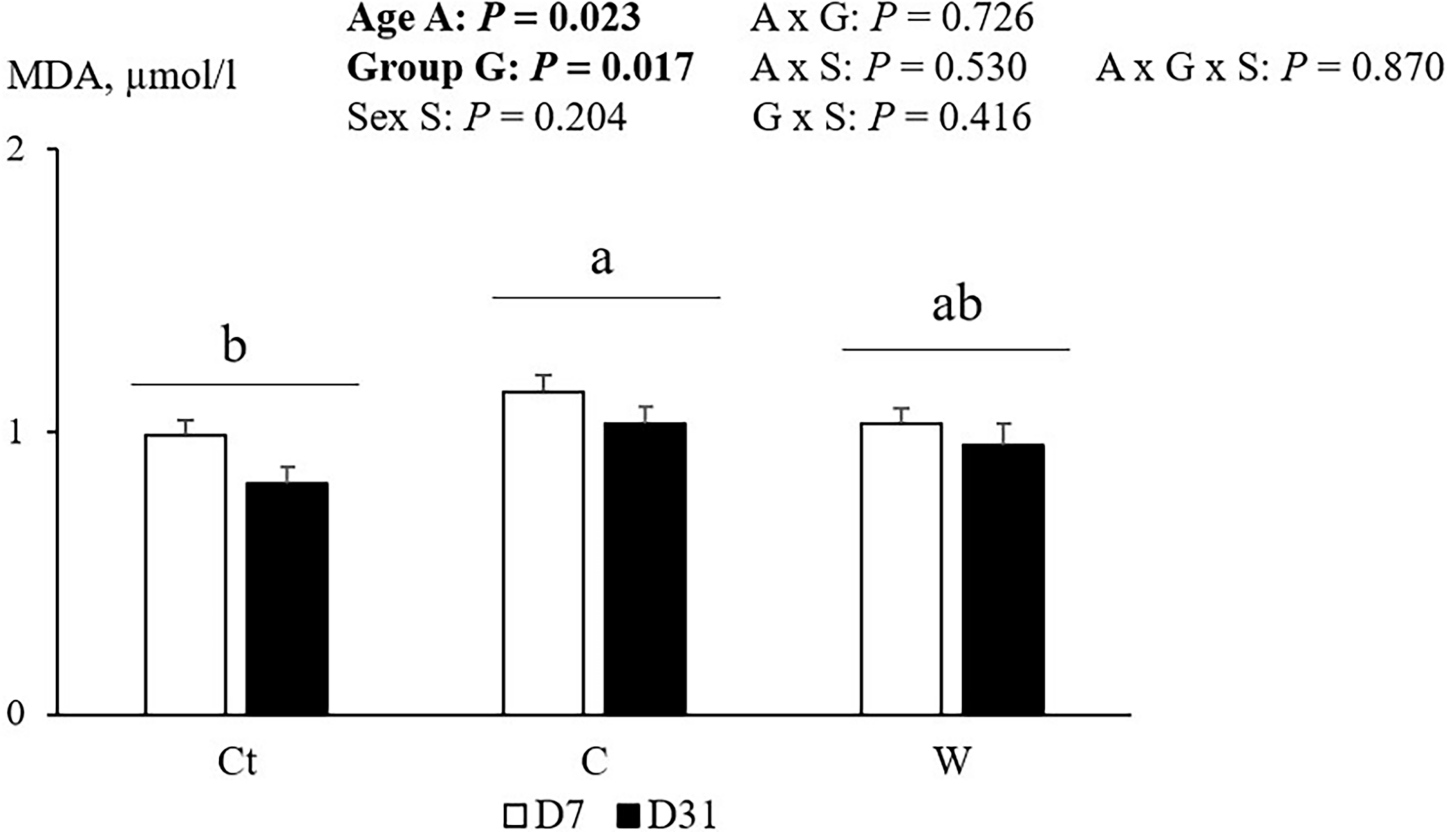
TBARS plasma index [expressed as Malondialdehyde concentration (MDA)] for E chickens (from eggs of breeder flock at the end of the laying period) at 7 and 31 days, depending on egg storage conditions and age of broilers (*n* = 8 animals, by sex per group). Ct: control group; C: cool storage; and W: warm storage. a, b: values not associated with common letters are statistically different ( *p* < 0.05).

Plasma TBARS results are reported in Figure 4.

There was no sex effect in this index, but age and group effects were observed, with no interaction between them. Indeed, the TBARS index decreased with age and was lower in the E-Ct group than in the E-C group, with the E-W group showing intermediate values.

**Figure 5:**
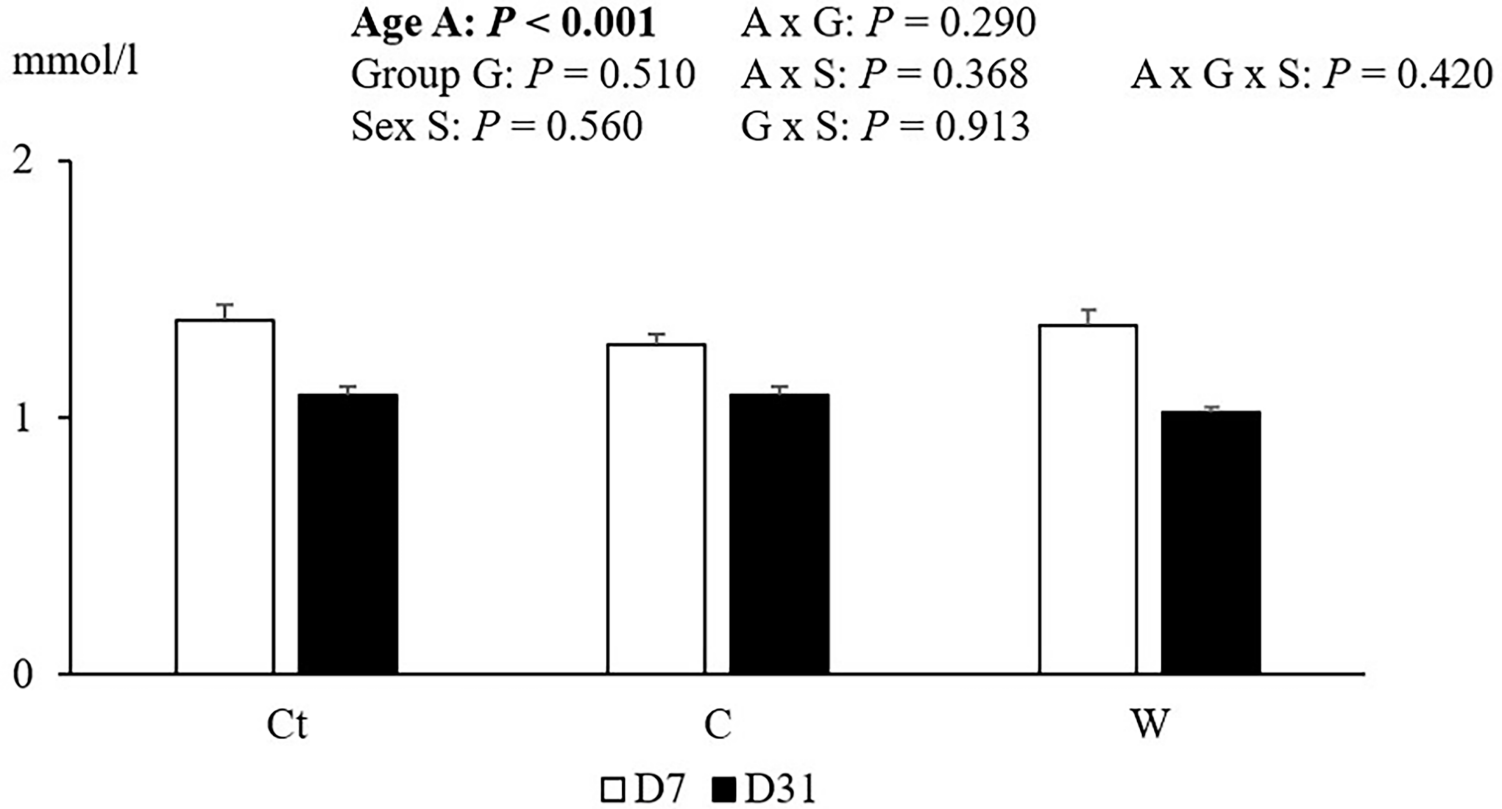
Total antioxidant power (TAS) of plasma for E chickens (from eggs of breeder flock at the end of the laying period) at 7 and 31 days, depending on egg storage conditions and age of broilers (*n* = 8 animals, by sex per group). Ct: control group; C: cool storage; and W: warm storage.

The effect of age, group and sex on TAS was also studied (Figure 5). Only an effect of age was observed for this indicator of blood antioxidant defences.

##### Metabolite Concentrations in the Plasma

There was no significant difference between groups for the plasma concentrations of glucose, uric acid and triglycerides. However, we observed an effect of age (d31 > d7, *p* = 0.011 for TG and d31 < d7 for AU) and sex for TG (male > female; *p* = 0.002).

## Discussion

Whether eggs came from young or old breeder flocks, pre-incubations and cool treatment during egg storage both resulted in reduced embryo mortality, which led to a subsequent decrease in the number of unhatched eggs and unhatched chicks, consequently increasing the number of hatched chicks. Short-, medium- and long-term effects of pre-incubations and cool treatment are discussed hereinafter.

### Short-Term Effects: Egg Weight Loss, Albumen pH, Embryo Development and Embryo Mortality

The detrimental effects of prolonged storage are mostly due to reduced albumen quality (Brake et al., 1997; Elibol and Brake, 2008) and the impairment of yolk membrane integrity, as the pH of the albumen increases with storage duration. Embryo viability depends on the total number of viable cells, the pH of the embryo’s micro environment and the embryo’s development stage (Reijrink et al., 2008).

In our study, storage at an average temperature of 11.6°C (vs. 18.3°C) appears to have limited the weight loss and increase in pH of eggs in group C compared to Ct. These results are in accordance with Hamidu et al. (2018), who showed that a lower storage temperature (18°C vs. 25°C) decreased egg weight loss, and Özlü et al. (2021), who observed a lower albumen pH with storage temperatures of 12 or 15°C rather than 18°C. Williams (1992) proposed that storage temperature should be maintained below 10°C in order to reduce the decline in albumen viscosity, but our study shows that an average of 11.6°C may be sufficient.

As expected, the pre-incubations conducted in our study on W eggs on days 6 and 10 after oviposition accelerated embryonic development from about stage EGX to XII, without however exceeding the critical stage of EGXIII (Reijrink et al., 2008). In contrast, embryo staging remained stable in other groups of eggs. A similar effect of pre-incubation on embryonic development was also reported in previous studies (Fasenko et al., 2001; Reijrink et al., 2009, 2010; Bakst et al., 2016; Özlü et al., 2021).

The number of eggs at stage 0 was high in Ct eggs: respectively, around 18.8 and 39.3% of eggs initially incubated in batches B and E. This result is in line with previous studies (reviewed in Bergoug et al., 2013a) showing that breeder flocks’ age negatively impacts egg fertilisation, but also the embryo’s ability to withstand prolonged storage periods in our study, pre-incubations and cool treatment decreased by more than 10% the proportion of eggs at stage 0 (in both B and E batches). This observation suggests that 10% of eggs discarded in Ct groups with the candling method could actually be considered as fertile eggs but with an embryo that died before 4 days of incubation. Thus, pre-incubations and cool treatment during storage preserved embryos from early mortality. Our results support previous studies (Pokhrel et al., 2018) suggesting that the first 3 days of incubation are critical for embryonic survival.

In Özlü et al. (2021), late embryo mortality decreased following pre-incubation treatments. In our study, middle and late embryo mortalities were generally much lower in all groups but were still impacted by pre-incubations and cool treatment in B eggs (until the 17th day of incubation). For E eggs, embryo mortality was probably less impacted at later stages because of the higher number of early mortalities (the weakest embryos were already dead at stage 0).

Regardless of the age of breeder flocks, there were no effects of pre-incubations and cool treatments on embryo mortality after 18 days of incubation, as there was no significant effect on embryo mortality at stage 4, nor on chick mortality at hatching.

### Medium-Term Effects: Hatching Results, Proportion of First Grade Chicks, Late Hatching, Hatching Body Weight, Vitellus Weight and Sex Ratio

The beneficial effects of pre-incubations and cool treatment on embryo mortality helped improve hatching results, increasing the hatching rate by more than 21% for B eggs and more than 13% for E eggs.

The better hatchability observed in our study with cool treatment during egg storage—whether the eggs were from young or old breeder flocks—is in accordance with Pokhrel et al. (2018), who compared the effects of a temperature of 12°C vs. 18°C on eggs stored from 7 to 28 days (these eggs came from 32- to 63-week-old breeder flocks). They found lower rates of early apoptosis with a lower temperature and persistence of the embryonic cytoarchitectural properties.

Damaziak et al. (2018) also found better hatchability rates with pre-incubations, but more significantly in older groups, which is in contrast to other studies (Gucbilmez et al., 2013; Ebeid et al., 2017; Özlü et al., 2021) where the increase in hatchability of fertile eggs was higher in eggs from young flocks than in eggs from older flocks. Similarly, the positive effect of pre-incubations on hatchability was more pronounced in our experiment for batch B (from 62% in Ct to 84% in W) than for batch E eggs (from 45% in Ct to 61% in W). This is probably due to the higher number of infertile eggs in older flocks that could not be changed whatever the storage conditions.

In Reijrink et al. (2009), pre-incubations had no effect on hatchability when the storage time was shorter than 7 days and were found to be both detrimental and beneficial when the storage period was prolonged. The reasons for this discrepancy were not clear. For instance, in Reijrink et al. (2010), the pre-incubation of eggs stored for 15 days had only a small effect on early embryonic mortality, and thus no effect on hatchability. In Fasenko et al. (2001), it improved hatchability from 72 to 82%. In both studies, the eggs were from breeder flocks whose hatchability is generally optimal [36 weeks old in Reijrink et al. (2010) and 32 in Fasenko et al. (2001)]. They both applied a single pre-incubation session at the beginning of storage. In our study, the better hatchability observed after several pre-incubations totalling 12 h during egg storage is in accordance with other studies such as Bakst et al. (2016) or Dymond et al. (2013), who found that pre-incubations restored hatchability to 84% by lowering both early and late embryo mortality. The total length of time of pre-incubation, whether applied at the beginning of the storage period or divided into various sessions throughout storage, thus appears to be of major importance.

Dymond et al. (2013) suggested that periodic pre-incubations on the blastoderm might reset the storage time perceived by the blastoderm and/or possibly provide an opportunity for blastodermal cells to perform basic cell functions and functions otherwise suppressed during egg storage. Regardless of the mechanisms involved, the beneficial effects of pre-incubations on hatchability appear to be due to the advancement of the developmental stage of the blastoderm. Fasenko et al. (2001) hypothesised that embryos between developmental stages EGXII and EGXIII are probably the most resistant to prolonged egg storage because they have already formed the hypoblast and are in a quiescent developmental stage.

Independently of the number of hatched chicks, the quality of hatched chicks—in terms of first/second grade ratio was improved in our study for both young and older breeder flocks after pre-incubations and cool treatment during egg storage. Within hatched chicks (Nh), cool treatment improved the proportion of first grade chicks even more than warm treatment, though significantly this only applies to batch B. However, although significant, the difference between the C and W groups was minor.

In several studies, chick quality was not affected by storage conditions. This was shown either in terms of the Pasgar score, body weight, chick length and yolk sac weight [in Pokhrel et al. (2018) comparing 12°C vs. 18°C storage conditions], or in terms of first/second grade ratio [in Özlü et al. (2021) comparing 12, 15 and 18°C storage temperatures, and in Damaziak et al. (2018) looking at pre-incubation effects], or in terms of chick lengths and yolk-free body mass [in Reijrink et al. (2009) applying pre-incubations]. However, all these studies found low percentages of second grade chicks (less than 1.5%), and chicks were of high quality in all storage conditions. This could explain the absence of an effect. Ebeid et al. (2017) found similar results to ours in that a pre-incubation period of 6 or 8 h improved the first grade proportion. This was not the case, however, for shorter pre-incubation times. This may partially explain the absence of an impact in other studies. Our results lead us to hypothesise that embryos resistant to long storage due to W or C treatments were of a higher quality and led to improved chick quality. They also suggest that as long as the embryo successfully recovers from diapause, the rest of its development continues normally. These findings are in agreement with Pokhrel et al. (2018), and with the fact that embryo mortality essentially occurs before the third day of incubation in Ct storage conditions.

While chicks are kept in the hatcher without water and feed, their body weight rapidly decreases due to dehydration and yolk use (Sklan et al., 2000; Bergoug et al., 2013b). Control (Ct) day-old chicks from batch B were heavier than W chicks and those from batch E were heavier than both W and C chicks. The proportional vitellus was also heavier in Ct chicks from batch E. This may be explained by the fact that Ct chicks hatched later: there were more wet chicks and unhatched chicks in Ct groups than in W or C groups in both B and E batches. In other words, pre-incubations (in batches B and E) and cool treatment (in batch E) foster precocious hatching. Many authors associate delayed hatching with egg storage. This delay could be explained by a delay in the initiation of embryogenesis after the start of incubation and/or by a decrease in the rate of embryonic development during incubation [reviewed by Nasri et al. (2020)]. The 8 h delay in the start of incubation of W eggs was efficient in our study, it being known that pre-incubation shortens the incubation period (Dymond et al., 2013). It also shows that the longer pre-warming (12 h) of C eggs to avoid condensation before the start of incubation was also efficient in shortening the incubation period or synchronising the hatching time (shorter hatching window). The later-hatched chicks in the Ct groups can be explained by a longer hatching window. On the contrary, W or C treatments made it possible to better synchronise hatching. Finally, W and C chicks had more time to resorb their vitellus into the abdominal cavity, thus making better use of energy resources for improved defences, increased robustness and limited pathogen entrance.

### Long-Term Effects: Mortality During Rearing, Body Weight, Relative Breast Muscle Weight and Oxidative Balance

In batch B, Ct chicks were still heavier than the C and W groups at 4 days of age, but there was no difference among groups from batch E. However, although significant, the batch B differences were small (2 g in body weight). After 4 days of age, pre-incubations and cool treatment during egg storage had no further visible consequences on post-hatching performance, i.e. mortality during rearing, body weight and relative breast muscle weight at the end of the rearing period (no interaction with sex). This is in accordance with Damaziak et al. (2018), who did not see any effect of pre-incubations on body weight until 42 days of life, but not with Dymond et al. (2013), who found a higher body weight following pre-incubations over the first 4 weeks post-hatching. However, in the latter study, mortality during the first week was not affected by pre-incubations.

The investigation of the oxidative balance conducted in poultry from batch E between 7 and 31 days of age was aimed to explore the metabolic effects of thermal treatments and on the redox balance during long-term effects. No effect of pre-incubations or cool treatment was shown on the plasma TAS. Although prestorage incubation has previously been shown to improve the antioxidative properties of chicks (Bakst et al., 2016; Ebeid et al., 2017), the present study did not reveal such an effect. Furthermore, the TBARS index—a potential marker of oxidative stress—was higher in C animals regardless of age. This raises the question of whether egg storage conditions perform a ‘selection’. Indeed, our results could indicate that by limiting the number of dead embryos, the cool treatment keeps more embryos alive, which overall would have a slightly less favourable redox balance than Ct animals. However, in our study, this trait was independent of the quality of hatched chicks, which was better for chicks from the C group than from the Ct group. To determinate a potential short-term effect of thermal treatments, the investigation of the oxidative balance needs further research, earlier in the embryo development or on hatched chicks.

Finally, our study revealed a decrease in the plasma TBARS index with age. This finding is consistent with the results previously obtained by Lin et al. (2004) between 0 and 14 days of age. Our results show that the plasma TAS was similarly affected by age. A decrease in plasma antioxidant defences was also reported between 0 and 21 days of age by Balogh et al. (2001).

### Sex Differences

The effect of thermal treatment during incubation on sex ratio was shown in other studies such as in Tzschentke and Halle (2009). In our study, the sex ratio of first grade chicks in the E batch was equally balanced and was not impacted by pre-incubations or cool treatment during egg storage. However, in the B batch, it appears that females were more sensitive to long storage because they represented only 43% of Ct first grade chicks, whereas the sex ratio at hatching was equally balanced when pre-incubations or cool treatment were applied. It is known that females hatch earlier than males (Van de Ven et al., 2011). The hatching window being longer in Ct—and leading to an interruption of the complete hatching process as hypothesised before—we should find more females than males in Ct, but the results for batch B showed the contrary. Mather and Laughlin (1976) reported that the difference in hatching time of both sexes disappears if the storage period is increased to 14 days. Wu et al. (2012) found that more female embryos died during incubation, especially in the early and middle stages. It is thus possible that embryo resistance and precocity in development are sex-dependent and should require further investigation. Indeed, the alteration in sex ratio induced by thermal stimulation could have a major impact on the commercial poultry industry, especially for layer hen producers, for whom male chicks have no commercial value and are culled upon hatching.

Whatever the group (C, W or Ct) or the batch (B or E), sex-related differences in chick/bird characteristics were observed. In both batches, day-old females were heavier than males with proportional vitellus also heavier in females (though this difference was significant only in E chicks). These differences were reversed from 11 days of age onwards. During rearing, males became heavier than females, as is commonly reported in various studies. Sexual dimorphism in breast yield is well documented in modern broilers (Zuidhof et al., 2014), but the number of birds used for breast muscle sampling was probably too low to reveal a statistical difference in breast muscle weight relative to body weight according to the sex.

## Conclusion

Whether eggs came from young or old breeder flocks, pre-incubations and cool treatment during egg storage both resulted in beneficial short- and medium-term effects on hatching results compared with conventional storage conditions. However, we did not find any major long-term effects during rearing of pre-incubations or cool treatment.

Changing storage conditions to obtain better hatchability rates is only beneficial to the industry and poultry if chick quality remains high. The benefits of pre-incubations or cool treatment on the hatching rates were very high in the present study compared with the number of incubated eggs, an outcome that is economically and practically relevant for commercial hatcheries.

In light of these results, and taking into account other practical constraints in hatcheries, pre-incubation or cool storage is interesting options to reduce the negative effects of long-term storage. Pre-incubation accelerates the developmental stage of the embryo, whereas cool storage limits weight loss and pH increase, thus contributing to a higher egg quality (albumen and vitelline membrane). These strategies have no adverse effects on chick robustness or on the health and performance of poultry throughout the rearing phase, at least in the specific case of suboptimal breeder flock ages explored in this study.

## Data Availability Statement

The raw data supporting the conclusions of this article will be made available by the authors, without undue reservation.

## Ethics Statement

The animal study was reviewed and approved by the Comité National de Réflexion Ethique sur l’Expérimentation Animale (National committee for ethics in animal testing) No. 016 at the French Ministry for Education, Higher Education and Research before data collection (number APAFIS#16128-2018071314183633 v1).

## Author Contributions

MG and JP designed the research studies. MG, JP, and RT carried out the experimental studies in collaboration with AK. AC designed and carried out the part on antioxidant mechanisms. PC, EC-A, and EC conducted the biochemical analyses. MG and AC analysed the data and performed the statistical analysis. MG, AC, and SR-G wrote the paper. All the authors read and approved the final manuscript.

## Funding

This research was undertaken under the QUALICOUV project, which received funding from the French Ministry for Agriculture, Food and Forestry (grant number CASDAR No. 1406-AAP2014), in the framework of UMT Sanivol.

## Conflict of Interest

The authors declare that the research was conducted in the absence of any commercial or financial relationships that could be construed as a potential conflict of interest.

## Publisher’s Note

All claims expressed in this article are solely those of the authors and do not necessarily represent those of their affiliated organizations, or those of the publisher, the editors and the reviewers. Any product that may be evaluated in this article, or claim that may be made by its manufacturer, is not guaranteed or endorsed by the publisher.
